# Clinical features and disease severity of Turkish FMF children carrying E148Q mutation

**DOI:** 10.1002/jcla.22852

**Published:** 2019-02-04

**Authors:** Fatma Aydın, Nilgün Çakar, Zeynep Birsin Özçakar, Nermin Uncu, Özge Başaran, Semanur Özdel, Elif Çelikel, Atilla H. Elhan, Fatoş Yalçınkaya

**Affiliations:** ^1^ Department of Pediatric Rheumatology Ankara University School of Medicine Ankara Turkey; ^2^ Department of Pediatric Rheumatology and Nephrology Ankara University School of Medicine Ankara Turkey; ^3^ Department of Pediatric Rheumatology and Nephrology Ankara, Child Health, Hematology and Oncology Education and Research Hospital Ankara Turkey; ^4^ Department of Pediatric Rheumatology Ankara, Child Health, Hematology and Oncology Education and Research Hospital Ankara Turkey; ^5^ Department of Biostatistics Ankara University School of Medicine Ankara Turkey

**Keywords:** E148Q, familial Mediterranean fever, MEFV mutation, pediatric, severity

## Abstract

**Background:**

Familial Mediterranean fever (FMF) is the most common hereditary monogenic autoinflammatory disease caused by mutations in the MEFV gene. It is controversial whether E148Q alteration is an insignificant variant or a disease‐causing mutation. The aim of this study was to evaluate the clinical features and disease severity of FMF patients carrying E148Q mutation.

**Methods:**

Files of FMF patients were retrospectively evaluated. Patients with at least one E148Q mutation were included to the study. The clinical characteristics and disease severity of the patients who were carrying only E148Q mutation were compared with the patients who were compound heterozygous for E148Q and homozygous for M694V mutation.

**Results:**

The study group comprised 33 patients who were homozygous or heterozygous for E148Q; 34 with compound heterozygous E148Q mutations and 86 patients who had homozygous M694V mutation. Patients who had only E148Q mutation were found to have the oldest mean age of disease onset and lowest mean disease severity score. Attack frequency and colchicine doses were lower in patients with only E148Q mutation as compared with the other two groups. The frequency of clinical findings such as fever, abdominal pain, arthralgia, and arthritis among the three groups was similar.

**Conclusion:**

Familial Mediterranean fever patients with only E148Q mutation are presenting with late‐onset and milder disease course despite having similar clinical findings as compared with patients who had other mutations. Finally, we imply that E148Q is a mutation and colchicine treatment should be given.

## 1 INTRODUCTION

Familial Mediterranean fever (FMF OMIM no. *608107) is an inherited autoinflammatory disorder characterized by recurrent short episodes of fever accompanied by sterile serositis including abdomen, chest, and joints.^1^ The disease most commonly affects Jewish, Armenian, Turkish, and Arab population with a prevalence of 1/1000‐1/500.^2^ However, patients of various ethnic origins are well documented after the discovery of the gene.^2^

Early and correct diagnosis is crucial, because often lifelong treatment with colchicine is needed to prevent attacks and renal amyloidosis which is the main complication of disease. The diagnosis of FMF is based on the presence of clinical symptoms and can be supported by genetic testing, but the absence of mutation does not exclude the disease.^3^ The gene responsible for FMF, designated MEFV, encodes a protein named as pyrin (alternatively marenostrin) which is primarily expressed in polymorphonuclear cells, cytokine‐activated monocytes, and synovial fibroblasts.^4^, ^5^ Pyrin has an important role in the inflammatory response by regulating caspase‐1 activation and processing mature IL‐1β.^6^

After identification of MEFV gene, the genotype was shown to be associated with a wide range of disease symptoms. The most common mutations responsible for FMF are in exon 10, such as M680I, M694V, M694I, and V726A.^2^ E148Q variation in exon 2 was first described as a mutation in 1998 and reported as the second most common alteration responsible for FMF after M694V mutation in non‐Ashkenazi Jewish patients.^7^ In other respects, some publications argued that E148Q is a functional polymorphism not a disease‐causing mutation.^8^, ^9^ However, none of these studies could rule out the possibility that E148Q has a phenotypic effect in several ethnic groups. Thus, the role of the E148Q mutation in the FMF pathogenesis still remains inconclusive.

The aim of this study was to evaluate the clinical features and disease severity of Turkish FMF patients carrying only E148Q mutation and compare the characteristics of this group who had severe exon 10 mutations.

## 2 MATERIALS AND METHODS

Files of FMF patients without amyloidosis who had been followed in Ankara University School of Medicine and Ankara Child Health and Hematology‐Oncology Education and Research Hospital Pediatric Rheumatology Departments in between January 2013 and January 2014 were retrospectively reviewed.

Demographic features, family history, clinical findings, laboratory tests, and genetic analysis of MEFV mutations were recorded. The diagnosis of FMF was based on the presence of pediatric clinical criteria.^3^ Disease severity was calculated according to Pras criteria.^10^

At least six predominant mutations (p.M694V, p.M680I, p.M694I, p.V726A, p.K695R, and p.E148Q) in the MEFV gene were studied in all patients. Direct sequencing of the PCR‐amplified fragments was used for screening the exon 10 of the MEFV gene, and PCR restriction fragment length polymorphism (RFLP) protocol was used for analyzing the p.E148Q mutation in exon 2.^11^, ^12^ Patients were divided into three groups according to the mutations. Group 1 included the patients who had only E148Q mutation, group 2 consisted of patients with compound heterozygous E148Q/Exon 10 mutations, and group 3 comprised M694V homozygous patients.

The study was conducted according to the Declaration of Helsinki and local regulations. Ethical approval for the study was obtained from the hospital's local ethics committee, and informed consent was obtained from all patients.

### 2.1 Statistical analysis

Results are given as mean ±SD, median (minimum‐maximum) or proportion as appropriate. Categorical variables were evaluated with chi‐square test. Comparison between three groups for the non‐normally distributed continuous variables was assessed by the Mann‐Whitney *U* test. A *P* value <0.05 was considered significant. All of the analyses were performed using SPSS version 15.

## 3 RESULTS

The study group included 153 FMF patients (86 females, 67 males) with a mean age of 12.1 ± 5.1 years. Group 1 had 33 (21.6%) patients 29 of whom had heterozygous and 4 homozygous E148Q mutation. Group 2 had 34 compound heterozygous patients with E148Q and exon 10 (31 M694V and 3 M680I) mutations. Group 3 had 86 (56,2%) patients with homozygous M694V mutation.

There was no significant difference with regard to the frequency of clinical findings. Myalgia and heel pain were most frequently encountered in groups 2 and 3, respectively (*P* < 0.05). Attack frequency before colchine treatment was significantly lower in group 1 as compared with the other two groups (*P* < 0.05). Final mean colchine doses and mean disease severity score were significantly lower in group 1 as compared with group 3 (*P* < 0.05).

None of the patients in group 1 had elevated acute phase reactants between attacks. All of the patients in group 1 had responded to colchicine treatment. The median acute phase reactants of patients in group 3 were higher in attacks and attack free periods as compared with the other two groups (data not shown).

Clinical and demographic characteristics, disease severity, and colchicine doses of FMF patients in three groups are shown in Table 1.

**Table 1:** Clinical and demographic characteristics, disease severity, and colchicine doses of FMF patients

Characteristics	Group I E148Q/‐,E148Q/E148Q n = 33	Group II E148Q/exon 10 n = 34	Group III M694V/M694V n = 86 (%)	*P* a, b, c	*P* a	*P* b	*P* c
Age (months) (median) (min‐max)	161 (24‐240)	125 (35‐279)	146 (16‐320)	0.443			
Male (%)	12 (36)	20 (58.8)	35 (40.7)	0.123			
Consanguinity (%)	8 (24.2)	5 (14.7)	27 (31.3)	0.166			
Family history of FMF (%)	12 (36.3)	24 (70.5)	55 (63.9)		0.005	0.007	0.492
Age at disease onset (month) (mean ±SD)	71.63 ± 55.34	51.03 ± 40.65	38.57 ± 32.55		0.188	0.002	0.105
Age at colchicine onset (month) (mean ±SD)	95.48 ± 49	73.24 ± 42.9	71.6 ± 42.3		0.066	0.013	0.749
Delay in diagnosis (month) (mean ±SD)	23.66 ± 22.32	21.64 ± 21.28	33.09 ± 33	0.272			
Attack frequency before colchicine (year) (mean ±SD)	15.69 ± 13.7	26.67 ± 18.77	27.36 ± 20.47		0.017	0.004	0.954
Fever (%)	29 (87.9)	29 (85.3)	78 (90.7)	0.683			
Abdominal pain (%)	30 (90.9)	28 (82.4)	77 (89.5)	0.472			
Chest pain (%)	6 (18.2)	15 (44.1)	22 (25.6)		0.023	0.39	0.048
Arthralgia (%)	16 (48.5)	18 (52.9)	42 (48.8)	0.910			
Arthritis (%)	7 (21.2)	5 (14.7)	20 (23.3)	0.583			
Myalgia (%)	4 (12.1)	11 (32.3)	7 (8.1)		0.049	0.5	0.001
Heel pain (%)	4 (12.1)	8 (23.5)	45 (52.3)		0.2	<0.001	0.004
Erysipelas like erythema (%)	3 (9.1)	4 (11.8)	5 (5.8)	0.526			
Protracted febrile myalgia (%)	0	0	2 (2.3)	0.454			
Vasculitis (%)	2 (6)	2 (5.8)	5 (5.8)	0.999			
Elevated attack free APR (%)	0	3 (8.8)	21 (24.4)		0.078	0.002	0.066
Pras severity score (mean ±SD)	5.97 ± 2.10	6.62 ± 2.01	7.81 ± 2.02		0.189	<0.001	0.003
Pras severity category (%)							
Mild	17 (51.5)	11 (32.4)	11 (12.8)	0.001			
Moderate	10 (30.4)	15 (44.1)	47 (54.7)				
Severe	6 (18.1)	8 (23.5)	28 (32.5)				
Colchicine dose, mg/day (mean±SD)	1.03 ± 0.30	1.02 ± 0.45	1.23 ± 0.43		0.655	0.046	0.010
Colchicine dose (mean±SD)							
mg/kg/day	0.026 ± 0.010	0.029 ± 0.014	0.034 ± 0.012		0.422	0.001	0.020
mg/m^2^/day	0.82 ± 0.21	0.88 ± 0.28	1.09 ± 0.27		0.527	<0.001	<0.001
Colchicine resistant patients (%)	0	1 (%2.9)	4 (%4.6)	0.44			

APR, acute phase reactant; FMF, familial Mediterranean fever.

a Group I versus group II

b Group I versus group III

c Group II versus group III.

## 4 DISCUSSION

It was first Aksentijevich et al^12^ who found a high carrier rate for *MEFV* mutations of 21% in 200 anonymous Ashkenazi Jewish DNA samples with being the most common mutation and suggested a heterozygote advantage in the Mediterranean area. In the Jewish Ashkenazi population, the carrier rate of the mutation E148Q is extremely high (1:10), yet FMF is rare in this community. The discrepancy between the prevalence of this mutation in the general population and among FMF patients shed doubt on the view that E148Q is a disease‐causing mutation. Some previous studies from different countries support this observation.^8^, ^12^, ^13^ Yılmaz et al^11^ reported that the carrier frequency of E148Q mutation was 12% in asymptomatic Turkish population who were admitted to the hospital and on the contrary the frequency was 3.5% in the patient group. However, a recent population‐based field study from Turkey revealed that 14.8% of 500 normal healthy individuals had MEFV mutations being the most common E148Q (4.8%) and the second M694V (3.2%).^14^

Despite the fact that the frequency of E148Q differs according to ethnicity, most studies from different ethnic communities and countries support the idea of having a phenotypic effect of E148Q.^15^, ^16^, ^17^, ^18^, ^19^ In recent studies, E148Q allele frequency was found 25% in Jewish, Arab, and Druze patients living in Israel^16^ and 20% in Turkish FMF patients living in Turkey.^18^ It was also reported that 34% Japanese FMF patients and 17% of Azeri Turk patients living in Iran had E148Q mutation.^15^, ^17^ The lowest frequency of E148Q mutation was found in Armenian FMF patients as 2%.^19^ This may indicate a varying severity of the disease. Other studies noted that the E148Q mutation was found to be associated with mild FMF symptoms, due to the lack of amyloidosis.^20^

In our study, no significant difference was found with regard to the frequency of clinical findings such as fever, abdominal pain, arthralgia, arthritis, and erysipelas like erythema among the patients with only E148Q, homozygous M694V and compound heterozygous E148Q/Exon 10 mutations. Chest pain seems to be less common with a lower statistical significance in the patients who had E148Q as compared to the other two groups. However, heel pain was found significantly lower rates in this group. In addition, all patients with only E148Q mutation responded to colchicine treatment and none had elevated acute phase reactants in between the attacks. These findings clearly revealed that E148Q is a disease‐causing mutation in Turkish patients with FMF. Similarly, Topaloglu et al^21^ reported no significant difference in the symptoms of attacks between patients with E148Q homozygotes and those with exon 10 mutations.

It was previously described that this mutation is milder and more frequent within the control groups than among patients.^8^, ^9^ On the other hand, E148Q was found to be the oldest of all the other mutations; it is estimated to be approximately 30 000 years of age.^22^ In the present study, 43% of patients were heterozygous, 51% were compound heterozygous (91% with M694V mutation), and only 6% were homozygous for E148Q mutation. Most of the E148Q mutations were shown to be heterozygous or compound heterozygous with Exon 10 mutations in the populations of the countries where FMF is prevalent.^20^ An interesting finding is that more than half of Druze FMF patients with E148Q mutation were homozygous.^16^

In our study, patients with only E148Q mutation had significantly older age at disease onset as compared to the patients with homozygous M694V and compound heterozygous E148Q/Exon 10 mutations. In addition, family history of FMF, attack frequency before colchicine treatment, median disease severity score, and final mean colchicine dose were lower in this group which indicates a milder phenotype in the patients with only E148Q mutation. Similar results were reported in Iranian Azeri Turks and in Turks from southeast of Turkey.^17^, ^23^, ^24^ Topaloglu et al^21^ from Turkey found that the severity of disease could be moderate/severe in patients with homozygous E148Q mutation in addition to high colchicine response. Another recent study revealed clinically severe disease in one fourth of heterozygous mutated Turkish patients for E148Q.^18^ All these results demonstrate that E148Q mutation usually produces a milder disease but may also have severe findings. In addition, although there has been no documented case of amyloidosis in homozygotes for E148Q, several compound heterozygote patients with E148Q/Exon 10 mutations have been demonstrated.^20^, ^25^, ^26^ Previous studies suggested the effect of environment on the severity of FMF and development of amyloidosis which is related to country of recruitment. This country context includes regional and ethnic factors in addition to the social and economic development. Poverty, early and repeated exposure to pathogens was thought to trigger attacks and provide ongoing inflammation in the patients living in high‐risk countries.^27^, ^28^

The retrospective nature of the study and low number of patients carrying homozygous E148Q mutations could be a relative limitation for our study. Another limitation of the study is that relatively rare mutations that cause disease have not been investigated.

**In conclusion,** patients with only E148Q alteration present with late‐onset and usually milder disease course despite having similar clinical findings and well response to colchicine therapy as compared with patients who had other mutations. Thus, it should be regarded as a disease‐causing mutation. As family history of FMF, attack frequency before colchicine treatment, median disease severity score, and final mean colchicine dose were lower in the patients who bear E148Q mutation high level of awareness of all clinicians working in countries with a high prevalence of FMF is required. We recommend colchicine treatment in those patients who are living in high‐risk communities. In addition, siblings should be questioned about the symptoms of FMF and molecular diagnosis be performed when clinical signs appear.

## CONFLICTS OF INTEREST

None.
